# Bio-Based Alginate Films Incorporating Bacterial Nanocellulose and Grape Seed Extract for Enhanced Food Packaging

**DOI:** 10.3390/polym17192564

**Published:** 2025-09-23

**Authors:** Urška Vrabič-Brodnjak, Tina Ružič

**Affiliations:** Department of Textiles, Graphic Arts and Design, Faculty of Natural Sciences and Engineering, University of Ljubljana, Aškerčeva 12, 1000 Ljubljana, Slovenia; tinckaruzic@gmail.com

**Keywords:** fruit packaging, sustainability, material analysis, natural extracts

## Abstract

The development of sustainable, active food packaging materials is essential for reducing plastic waste and improving food preservation. This study investigated the fabrication and characterization of bio-based films composed of sodium alginate (Na-alginate), bacterial nanocellulose (BNC), and grape seed extract (GSE) as a natural antioxidant. Films were prepared via casting solutions with 2% Na-alginate, 1% and 2% of BNC, glycerol as a plasticizer, and varying GSE concentrations (0, 0.5, 1, and 2% *w*/*w*). The films’ physicochemical properties, including thickness, mechanical strength, water vapor permeability, antimicrobial and antioxidant activity (DPPH assay), were evaluated. To assess practical applicability, blueberries were packaged in these films and stored at 4 °C for four weeks, with spoilage, weight loss, and visual quality monitored. The results demonstrated that GSE significantly enhanced the films’ antioxidant capacity, with 1% GSE achieving an optimal balance between mechanical integrity and bioactivity. Blueberries packaged in GSE-enriched films exhibited lower spoilage, reduced weight loss, and maintained better visual appearance compared to controls. These findings suggest that Na-alginate/BNC/GSE films possess potential as biodegradable active packaging materials for extending the shelf life of perishable fruits.

## 1. Introduction

The increasing global production of plastic waste, caused by rising consumer demand and increasing industrial activity, has become a major environmental challenge [1,2,3]. Therefore, there is an urgent and growing need for sustainable, biodegradable, and environmentally friendly packaging solutions that can effectively replace conventional plastics in food packaging and other applications [2,3]. In response to this global concern, extensive research has been conducted to develop biopolymers and natural materials from renewable resources that offer potential pathways to environmentally friendly packaging options that are in line with the principles of sustainability and the circular economy [4,5,6,7,8]. Promising candidates for sustainable packaging materials include polysaccharides, a diverse class of natural biopolymers derived from plants, algae, and bacteria. These polymers are inherently biodegradable, biocompatible, and non-toxic, and many of them possess intrinsic film-forming capabilities that make them particularly suitable for food-contact applications [9,10,11,12]. Among polysaccharides, sodium alginate (Na-alginate) has attracted considerable attention due to its unique physicochemical and functional properties [12,13,14]. One of the most distinctive features of sodium alginate is its ability to undergo ionic gelation in the presence of divalent cations, such as Ca^2+^, forming so-called “egg-box” junctions where G-blocks interact cooperatively with calcium ions. This property underlies alginate’s wide use in biomedical, pharmaceutical, and food-related applications as it enables the formation of stable hydrogels and flexible thin films. In the context of food packaging, sodium alginate films exhibit several attractive characteristics. They are transparent, flexible, odorless, and safe for consumption (generally recognized as safe) while also offering biodegradability and compostability, making them environmentally friendly alternatives to petroleum-based plastics. Furthermore, their hydrophilic nature facilitates compatibility with a variety of natural additives, including plasticizers, nanofillers, and bioactive compounds, enabling the design of multifunctional packaging systems. Despite these advantages, sodium alginate films face significant limitations that restrict their direct commercial application. Pure alginate films generally exhibit low mechanical strength, making them brittle and prone to cracking under stress [15,16,17,18]. In addition, their high moisture sensitivity results in poor water vapor and gas barrier properties, limiting their ability to prevent dehydration or oxidative spoilage of packaged foods. Moreover, alginate films have limited resistance to flavor and aroma transfer, which reduces their effectiveness in maintaining food sensory quality [19,20,21]. To overcome these drawbacks, numerous modification and reinforcement strategies have been investigated. The incorporation of nanostructured biopolymers such as BNC has proven effective in enhancing tensile strength and barrier properties through polymer–nanofiber interactions [22,23]. Therefore, chemical cross-linking, blending with other polysaccharides (e.g., chitosan, pectin, and starch), and the addition of lipids and proteins have also been explored to reduce water sensitivity and improve functional stability [24,25]. Namely, BNC is a nanostructured form of cellulose produced extracellularly by certain bacterial strains, most notably *Komagataeibacter xylinus* (formerly *Acetobacter xylinum*), through the fermentation of carbohydrates [25,26,27]. The unique nanofibrillar architecture gives BNC a set of remarkable physicochemical properties. It exhibits very high crystallinity (typically above 80–90%), exceptional tensile strength, and a high degree of polymerization compared to plant cellulose. The abundant hydroxyl groups on its surface enable strong hydrogen bonding, which facilitates excellent interfacial interactions when combined with other biopolymers [23,24,25]. In addition, the high surface area and water-holding capacity of BNC make it versatile for use in composite materials, while its inherent biodegradability and non-toxicity support its application in environmentally friendly packaging systems [27,28,29]. When incorporated into polysaccharide matrices, such as sodium alginate, BNC functions primarily as a reinforcing nanofiller [29,30,31,32]. Its rigid crystalline domains improve stress transfer and reduce polymer chain mobility, leading to films with significantly enhanced tensile strength and reduced elongation at break. At the same time, the well-dispersed nanofibrils increase the tortuosity of diffusion pathways, thereby reducing water vapor and gas permeability. This barrier improvement is particularly critical for food packaging, where moisture retention and oxidative stability determine product shelf life. Its ability to form strong hydrogen bonds with other natural additives, such as phenolic compounds, proteins, or essential oils, facilitates the development of active packaging systems with tailored properties. For instance, when combined with antioxidant-rich GSE, BNC provides not only structural enhancement but also a matrix that stabilizes and retains the bioactive compounds, enabling a synergistic improvement in both mechanical and functional performance [33,34]. From a sustainability perspective, BNC is highly advantageous as it can be produced via microbial fermentation using renewable feedstocks, including agro-industrial byproducts and waste streams.

Active packaging, containing antioxidants, antimicrobials, or both, can significantly delay spoilage processes, such as oxidation and microbial proliferation, thus prolonging the freshness and edibility of food. Among natural antioxidants, grape seed extract has attracted considerable attention due to its high content of polyphenols, especially proanthocyanidins, which have been shown to have strong antioxidant and antimicrobial effects. The abundance of these compounds makes GSE an excellent additive for bio-based packaging materials designed to extend the shelf life of fresh produce and other perishable foods [35,36,37]. By integrating GSE into alginate BNC matrices, active packaging films can be produced that are able to inhibit oxidation reactions and microbial growth, ultimately improving food safety and quality [36,37,38]. Studies have shown that the incorporation of GSE can affect the transparency, mechanical strength, and water vapor permeability of the films [39,40]. While higher concentrations of GSE may increase opacity, potentially protecting light-sensitive foods from degradation, they can also impact the tensile strength and flexibility of the films. Beyond their structural role, GSE-infused films have demonstrated efficacy in inhibiting the growth of various microorganisms, including both Gram-positive and Gram-negative bacteria. This antimicrobial activity is attributed to the phenolic compounds present in GSE, which can disrupt microbial cell membranes and inhibit their growth [41,42]. Furthermore, the antioxidant properties of GSE contribute to the preservation of food quality by scavenging free radicals and preventing oxidative damage.

The aim of this research was to develop and characterize biodegradable, active packaging films based on sodium alginate, BNC, and GSE for the preservation of perishable fruits, using blueberries as a model system. The study seeks to leverage the synergistic properties of these components—Na-alginate for film-forming capability, BNC for mechanical reinforcement and barrier enhancement, and GSE for antioxidant and antimicrobial functionality—to create sustainable packaging materials with improved performance.

The objectives of this research were as follows:

- To fabricate composite films of Na-alginate reinforced with BNC and enriched with different concentrations of GSE.
- To evaluate the physicochemical, mechanical, and barrier properties of the prepared films.
- To assess the antioxidant and antimicrobial activities imparted by GSE within the film matrices.
- To investigate the effectiveness of the developed films in extending the shelf life, maintaining quality, and reducing spoilage of fresh blueberries during storage.
- To explore the potential of utilizing agro-industrial byproducts (grape seeds) and microbial biopolymers (BNC) in sustainable and circular economy-oriented food packaging solutions.

This research provides new insights into the potential of combining renewable biopolymers, nanostructured reinforcements, and natural bioactives to produce multifunctional, sustainable packaging materials. The use of alginate-based films reinforced with bacterial nanocellulose and enriched with natural antioxidants such as GSE is a promising route to sustainable, active packaging solutions.

## 2. Materials and Methods

### 2.1. Materials

Sodium alginate (biological source: brown algae; white powder, viscosity ≥2000 cP, 2% (25 °C)) was obtained from Sigma-Aldrich (St. Louis, MO, USA). Bacterial nanocellulose suspension (1% *w*/*w*) was produced in-house via fermentation using *Komagataeibacter xylinus* following established protocols from our previous research [1]. Grape seed extract powder, standardized to 95% polyphenols, was purchased from a commercial supplier (Sigma-Aldrich; USA). Glycerol (viscous liquid; molecular weight 92.09) was used as a plasticizer and purchased from Sigma-Aldrich (St. Louis, MO, USA). Fresh blueberries were sourced from a local market and used immediately after purchase.

### 2.2. Film Preparation

Film-forming solutions were prepared by dissolving 2% (*w*/*w*) sodium alginate in distilled water under magnetic stirring at room temperature (~23 °C) for 1 h until fully dissolved. The BNC suspension was incorporated once at 1% (*w*/*w*) and the second time 2% (*w*/*w*) into the 2% (*w*/*w*) of alginate solution, using an ultrasonic processor (Branson Ultrasonic SA, Urdorf, Switzerland). The ultrasonic processor was operating at 20 kHz for 5 min to ensure uniform dispersion. Glycerol was added at 15% (*w*/*w*) relative to the weight of alginate as a plasticizer and homogenized into the mixture. GSE was dissolved separately in distilled water and added at concentrations of 0 (control), 0.5, 1, and 2% (*w*/*w*) of the total solution, with stirring (30 min) performed until the solution was homogeneous. Films were cast by pouring 20 mL of each solution into polystyrene Petri dishes (90 mm diameter). The films were dried in an oven at 40 °C for 48 h. Once dried, the films were carefully peeled off and conditioned at controlled relative humidity of 50 ± 2% and temperature 23 ± 1 °C for 48 h prior to characterization (Table 1).

### 2.3. Film Analysis and Characterization

#### 2.3.1. Film Thickness and Porosity of the Films

Thickness of the films were measured at ten random points per film using a digital micrometer Mitutoyo, resolution 0.01 mm (Mitutoyo Corp., Hiroshima, Japan). Porosity was carried out using a Bendtsen instrument (Alat UJI, Jakarta, Indonesia). The measurement range was 0 to 5000 mL/min, with air flowing through a flat metal ring and the test sample. The pressure difference was recorded using the rotameter tube provided. Ten replicates were measured on each sample. At thickness and porosity results, standard deviation results are also presented.

#### 2.3.2. Mechanical Properties

Tensile properties were determined by a universal testing machine (Instron 5567, Instron, Norwood, MA, USA) in accordance with standard ASTM D882-12 [42]. Rectangular strips (10 mm × 60 mm) were mounted and subjected to tensile testing at a crosshead speed of 5 mm/min. Data were collected to derive tensile strength (TS) and elongation at break (EB).

#### 2.3.3. Water Vapor Permeability (WVP)

WVP was assessed gravimetrically following standard ASTM E96-16 [43]. Film samples were sealed over cups containing water and placed in a controlled humidity chamber for 24 h (Binder GmbH; Tuttlingen, Germany). The weight gain was recorded over time, and WVP was calculated using the standard procedure as described in the above-mentioned standard.

#### 2.3.4. Antioxidant Activity

Antioxidant activity was determined via the DPPH radical scavenging assay. Film extracts were prepared by soaking 1 cm, 2-film pieces in 10 mL of methanol for 24 h. The methanolic extracts were reacted with DPPH solution, and absorbance at 517 nm was measured with a spectrophotometer (Shimadzu UV-2600 spectrophotometer, Shimadzu Corp., Kyoto, Japan) to quantify radical-scavenging activity, expressed as percentage inhibition relative to controls. Each sample was measured 4 times, and the results are averaged. Free DPPH radical scavenging rate was calculated using this equation:

(1) $$\kappa_{DPPH}=\frac{A_{C}-A_{S}}{A_{C}}\times 100\%$$

#### 2.3.5. Total Phenolic Content (TPC)

TPC was quantified using the Folin–Ciocalteu method [44]. Extracts were reacted with Folin–Ciocalteu reagent and sodium carbonate, and absorbance was measured on the instrument UV–VIS spectrophotometer (Shimadzu UV-2600, Shimadzu Corp., Kyoto, Japan). The standard curve was drawn with the concentration of aqueous gallic acid as abscissa, and its absorbance was ordinate at 765 nm. The following equation was obtained:

(2) $$y=0.117x+0.0171(R^{2}=0.9995)$$

Each sample was measured in triplicate, and average values are presented in the results. The results are expressed as mg of gallic acid equivalents (GAEs) per gram of dry film, GAEmg/DWg.

#### 2.3.6. Antimicrobial Activity

The antimicrobial activity of composite packaging films was evaluated by the disc diffusion method against *E. coli* (ATCC 25922) and *Staphylococcus aureus* (ATCC 25923). The bacterial suspensions were adjusted to 0.5 McFarland standard and spread on Mueller–Hinton agar plates. Sterile round pieces of the films (6 mm diameter) were aseptically placed on the inoculated agar surface, together with positive control discs and solvent-free negative controls. After incubation at 35 ± 2 °C for 24 h, the diameter of the inhibition zones around the film samples was measured, and the antimicrobial activity was expressed as mean ± SD of three replicates.

#### 2.3.7. Statistical Analysis

Film thickness, porosity, and mechanical property measurements were performed in 10 probes at each sample. WVP was carried out in parallel. Antimicrobial activity was performed on triplicates. Data were analyzed using one-way ANOVA at a 95% confidence level (*p* < 0.05) in Microsoft^®^ Excel 2016 with the Data Analysis ToolPak. Experiments were performed following applicable standards, and the findings are presented as mean ± standard deviation.

### 2.4. Blueberry Packaging and Storage

Blueberries (2 per sample) were packaged in pouches cut from the prepared films and sealed on three sides with a heat sealer (Keiser Kraft GmbH; Stuttgart, Germany). Stored at 4 °C for two weeks. The number of blueberries was limited to two, as adding more would have exceeded the pouch capacity and risked opening during storage.

Weekly evaluations included the following:

- Visual Quality Assessment: Inspection for color changes, mold growth, and other visual spoilage indicators.
- Weight Loss: Weighed before and after storage; expressed as a percentage of initial weight.

## 3. Results and Discussion

Film-forming solutions based on sodium alginate were successfully prepared with the incorporation of BNC and GSE as functional additives. After drying and conditioning, the obtained films were uniform, transparent, and easy to peel from the casting surface, which indicated good miscibility of the components and homogeneous dispersion of BNC within the alginate matrix.

### 3.1. Film Thickness and Mechanical Properties

The thickness and mechanical properties of the films, namely, tensile strength and elongation at break, were significantly influenced by the concentration of BNC and GSE (Table 2). All samples showed porosity values that were below the detection limit of the applied analytical method and are, therefore, indicated as “zero” in Table 2. This does not mean that there are absolutely no pores but rather that the residual porosity and air permeability were too low to be quantitatively resolved with the method used. The results can be explained by the very dense and compact structure of the bacterial nanocellulose (BNC), which forms an interwoven nano-fibrillar network with minimal voids. This structure is further stabilized by sodium alginate, which acts as a continuous, film-forming polymer and improves the integrity of the barrier. The addition of grape seed extract likely contributes to the additional filling of nanoscale or submicron voids within the matrix. Taken together, these effects result in films with negligible air permeability and apparent porosity values below the measurable threshold.

Compared to the control films (without GSE), the addition of BNC improved the tensile strength of the alginate films in a concentration-dependent manner. At 1% BNC, the tensile strength increased from 25.2 ± 1.4 MPa (1BNC0) to 30.1 ± 1.5 MPa (1BNC1) and further to 31.7 ± 1.5 MPa (1BNC2). A similar trend was observed for the 2% BNC series, where the tensile strength increased from 26.8 ± 1.2 MPa (2BNC0) to 39.7 ± 1.0 MPa (2BNC2). These results demonstrate the reinforcing effect of BNC, which is due to the high crystalline, large surface area, and strong hydrogen bonding of the nanocellulose, which enables effective stress transfer between the alginate chains and the cellulose nanofibrils. However, the improvement in strength was accompanied by a reduction in elongation at break. The control film 1BNC0 exhibited the highest elongation (15.8 ± 0.9%), while the elongation progressively decreased with increasing BNC loading, reaching 12.4 ± 0.5% at 2% BNC. Similarly, elongation decreased from 50.8 ± 2.4% (2BNC0) to 42.5 ± 2.3% (2BNC2) for the 2% BNC series. This inverse relationship between tensile strength and elongation suggests that BNC reduces the flexibility of the polymer chains by introducing rigid domains and restricting molecular mobility. The addition of GSE also influenced film properties. At lower GSE levels (0.5–1%), the tensile strength increased compared to the GSE-free controls. For example, the tensile strength of the 1% BNC films increased from 25.2 ± 1.4 MPa (1BNC0) to 28.5 ± 1.2 MPa (1BNC0.5) and 30.1 ± 1.5 MPa (1BNC1). The tensile strength of the 2% BNC films also improved from 26.8 ± 1.2 MPa (2BNC0) to 39.7 ± 1.0 MPa (2BNC1). The highest tensile strength was achieved with 2BNC2, indicating that BNC and GSE acted synergistically in reinforcing the polymer network. This effect can be attributed to additional hydrogen bonding and possible interactions between the GSE polyphenols and the hydroxyl groups of alginate and BNC, improving interfacial adhesion and reducing microstructural defects.

On the other hand, the addition of GSE also reduced the elongation at break, especially at higher concentrations. Thus, the elongation decreased from 15.8% (1BNC0) to 12.4% (1BNC2). A similar reduction was observed in the 2% BNC group. These results suggest that while GSE helps to strengthen the film, it also increases brittleness, which is likely due to the formation of a stiffer polymer network with limited capacity for plastic deformation. The results demonstrate that both BNC and GSE improved the tensile strength of alginate-based films, with the greatest enhancement observed at the highest concentrations of both additives. However, these improvements were achieved at the expense of flexibility, as indicated by the consistent reduction in elongation at break. Such a trade-off between strength and extensibility is typical in polysaccharide-based nanocomposite films, where the incorporation of rigid nanofillers and polyphenolic compounds enhances load-bearing capacity but restricts chain mobility.

### 3.2. Results of Water Vapor Permeability (WVP)

The water vapor permeability (WVP) of the alginate-based films was significantly affected by both BNC and GSE incorporation (Table 3). In general, the inclusion of BNC led to a reduction in WVP values, indicating improved barrier performance against water vapor transmission.

For the films without GSE, WVP decreased with increasing BNC concentration. In the 1% BNC series, the WVP value dropped from 4.3 g·mm/m^2^·h·kPa (1BNC0) to 2.8 g·mm/m^2^·h·kPa (1BNC2). A similar decreasing trend was observed in the 2% BNC series, where WVP was reduced from 4.3 g·mm/m^2^·h·kPa (2BNC0) to 2.9 g·mm/m^2^·h·kPa (2BNC2). These results confirm the role of BNC as an effective barrier-enhancing component. The reduction in WVP can be attributed to the tortuous pathway effect created by well-dispersed nanocellulose fibrils within the alginate matrix, which increases the diffusion path length for water vapor molecules and thus hinders their permeability. At the same trend, the incorporation of GSE further contributed to reducing WVP, particularly at intermediate concentrations. This improvement is likely related to the polyphenolic nature of GSE, which may form additional hydrogen bonds with alginate and BNC, thereby enhancing the compactness of the polymer network and reducing free volume available for water vapor diffusion. The incorporation of GSE further contributed to reducing WVP, particularly at intermediate concentrations. The lowest WVP values were recorded for films with the highest concentrations of both BNC and GSE, confirming that the combined effect of BNC and GSE leads to superior barrier properties. These results indicate that the addition of nanocellulose and GSE not only reinforces the films mechanically but also enhances their functional barrier performance, which is particularly advantageous for potential applications in food packaging, where moisture resistance is critical.

### 3.3. Antioxidant Activity and Total Phenolic Content

**Figure 1:**
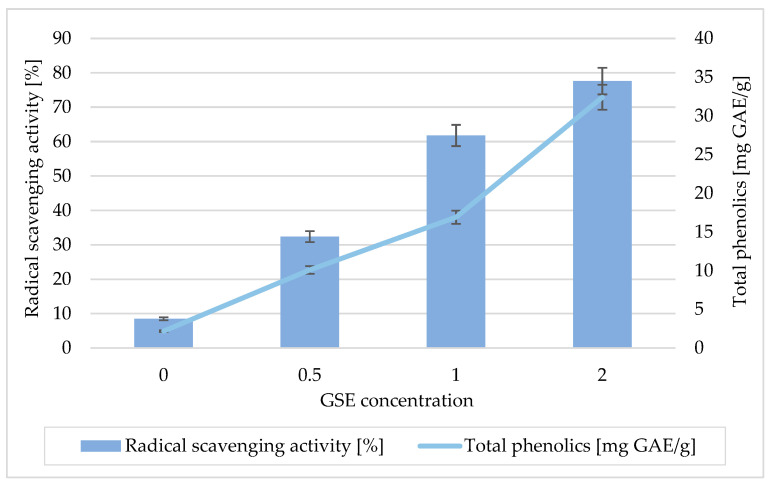
Results of radical scavenging activity and total phenolics content of GSE concentration.

The primary functional goal of incorporating GSE was to enhance antioxidant capacity. The antioxidant potential of the alginate-based films was assessed in terms of radical scavenging activity and total phenolic content (TPC) (Figure 1). The results demonstrated a strong dependence on the concentration of grape seed extract (GSE) incorporated into the film matrix.

Films without GSE showed negligible antioxidant activity (8.1%) and very-low phenolic content (4.6 mg GAE/g), indicating that the sodium alginate–BNC–glycerol matrix does not contribute significantly to the antioxidant response.

The addition of GSE led to a progressive increase in both free radical scavenging activity and phenolic content in a concentration-dependent manner. At a GSE loading of 0.5%, free radical scavenging activity increased to 31.7%, accompanied by an increase in total phenols to 11.9 mg GAE/g. A further increase to 1% GSE almost doubled the activity (61.8%), with total phenols reaching 18.2 mg GAE/g. The highest GSE concentration (2%) showed the strongest effect, with a free radical scavenging activity of 76.4% and a phenolic content of 32.1 mg GAE/g. These results show a strong positive correlation between total phenolic content and antioxidant activity and confirm that the antioxidant potential of the films is primarily due to the phenolic compounds in GSE. The observed trend is consistent with the well-documented antioxidant properties of grape seed polyphenols, particularly proanthocyanidins, catechins, and gallic acid derivatives, which act as effective hydrogen donors and free radical scavengers. Similar results have been reported for other polysaccharide-based films enriched with plant extracts, where the incorporation of polyphenols significantly improved the free radical scavenging efficiency in a concentration-dependent manner. Of note, varying the BNC content (1% vs. 2%) had no significant effect on antioxidant activity, suggesting that the composition of the matrix had little effect on the release or activity of the phenolic compounds. Instead, the antioxidant properties of the films were primarily determined by the concentration of GSE contained.

These results confirm the successful development of composite packaging films with adjustable antioxidant functionality. By increasing the GSE loading, the films achieved both high free radical scavenging activity and increased phenolic content, which, in combination with the proven antimicrobial performance, emphasizes their potential for use as active packaging materials designed to extend shelf life and improve the safety of perishable foods.

### 3.4. Results of Antimicrobial Activities of Prepared Samples

BNC forms a highly entangled nanofibrillar network with extensive hydrogen bonding, which creates a dense and compact matrix. This structure can physically entrap polyphenolic compounds and reduce their diffusion rate through the film. In addition, hydroxyl groups on BNC and alginate can form hydrogen bonds with the hydroxyl groups of GSE polyphenols (e.g., catechins, proanthocyanidins, and gallic acid derivatives), which may further immobilize these compounds within the polymer network. Such interactions could explain why antioxidant activity in solution assays appears unaffected by BNC concentration, while antimicrobial assays—which rely on local diffusion of active compounds at the film–microbe interface—are more sensitive to diffusion constraints. Similar phenomena, where polyphenol–polysaccharide interactions modulate release kinetics and bioactivity, have been reported in other biopolymer-based active films [36,41].

The antimicrobial performance of the composite films produced was evaluated using the disc diffusion method against *Escherichia coli* and *Staphylococcus aureus* (Table 4). Films without grape seed extract (1BNC0 and 2BNC0) showed no measurable inhibition zones, with a diameter corresponding only to the disc size (~6 mm), suggesting that the BNC–glycerol matrix itself has no intrinsic antimicrobial activity.

Following the addition of grape seed extract (GSE), distinct zones of inhibition were observed, the diameter of which increased in proportion to the GSE concentration. Moderate activity was observed at a GSE loading, of 0.5, with inhibition zones of 8.0–8.4 mm for *E. coli* and 9.0–9.2 mm for S. aureus. A further increase in GSE concentration to 1% significantly enhanced the antimicrobial effect, resulting in inhibition zones of 12.4–12.8 mm and 13.6–14.4 mm, respectively. The strongest activity was obtained at 2% GSE, with inhibition zones of more than 18 mm against *E. coli* and almost 20–21 mm against S. aureus. These results confirm a concentration-dependent antimicrobial activity of GSE when incorporated into BNC-based films. Comparison between the two-film series (1% vs. 2% BNC) revealed no significant differences in antimicrobial performance at equivalent GSE concentrations. Slightly smaller inhibition zones were observed in the 2BNC samples compared to the 1BNC samples, especially at higher GSE loads (e.g., 18.1 ± 0.5 mm vs. 19.3 ± 0.3 mm against *E. coli* at 2% GSE). This suggests that a higher BNC content may hinder the diffusion of the active ingredients into the agar medium, thereby slightly reducing their apparent antimicrobial efficacy. In terms of bacterial susceptibility, *S. aureus* was consistently more sensitive to the films than *E. coli*, showing ~1–1.5 mm larger inhibition zones at all GSE concentrations. This observation consists of the structural differences between Gram-positive and Gram-negative bacteria. The thick peptidoglycan layer of *S. aureus* can be more easily penetrated by the polyphenolic compounds contained in GSE, whereas the outer membrane of *E. coli* can act as a barrier and reduce susceptibility.

The results show that the antimicrobial properties of the films are primarily determined by the GSE content, while the BNC concentration plays a secondary role, by slightly influencing the diffusion but not changing the overall trend. The strong inhibition observed at 2% GSE indicates that these composite films have the potential to be used as active food packaging materials, especially against Gram-positive spoilage bacteria and pathogenic bacteria.

### 3.5. Blueberry Packaging and Storage Evaluation

Blueberries were selected as a representative model due to their high moisture content, rapid perishability, and susceptibility to oxidative damage. Similar protective effects may be expected for other high-moisture foods such as strawberries, tomatoes, or fresh-cut apples, where surface moisture facilitates the release of polyphenols from the film. In contrast, for lipid-rich foods (e.g., cheese, nuts, or processed meats), the impact may be less pronounced as the predominantly polar phenolics in GSE have lower solubility in nonpolar matrices.

Blueberries packaged in films containing 2% GSE showed the least weight loss (3.8%), compared to unwrapped (control) berries (11.3%) and berries wrapped in pure Na-alginate/2% BNC films (3.8%). Notably, the presence of GSE contributed to reducing moisture loss, possibly through enhanced barrier properties and antioxidant effects that slow down metabolic respiration and water migration (Table 5).

**Figure 2:**
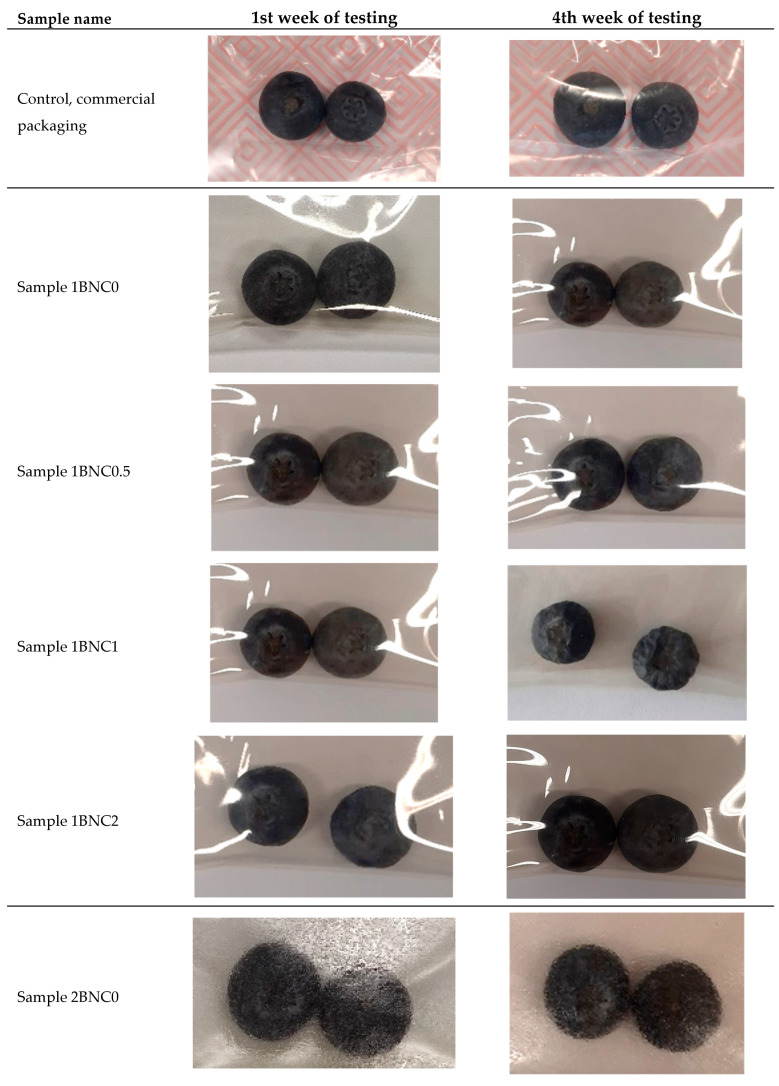

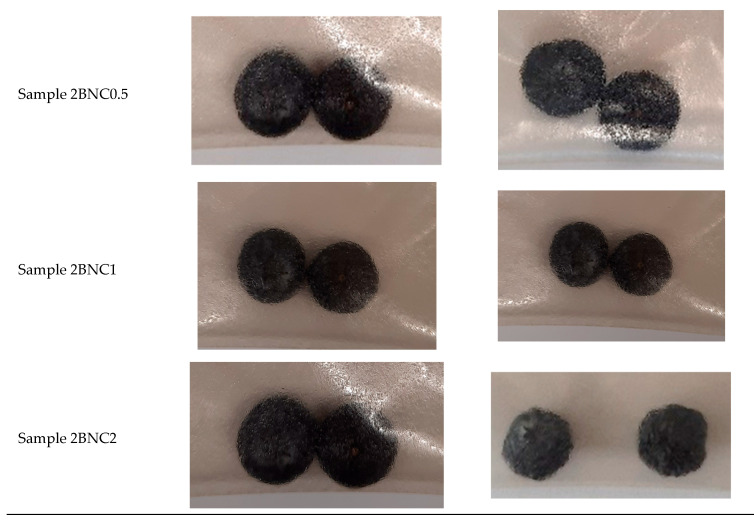
Results of packed berries in prepared films, with different concentrations of BNC and GSE.

**Figure 3:**
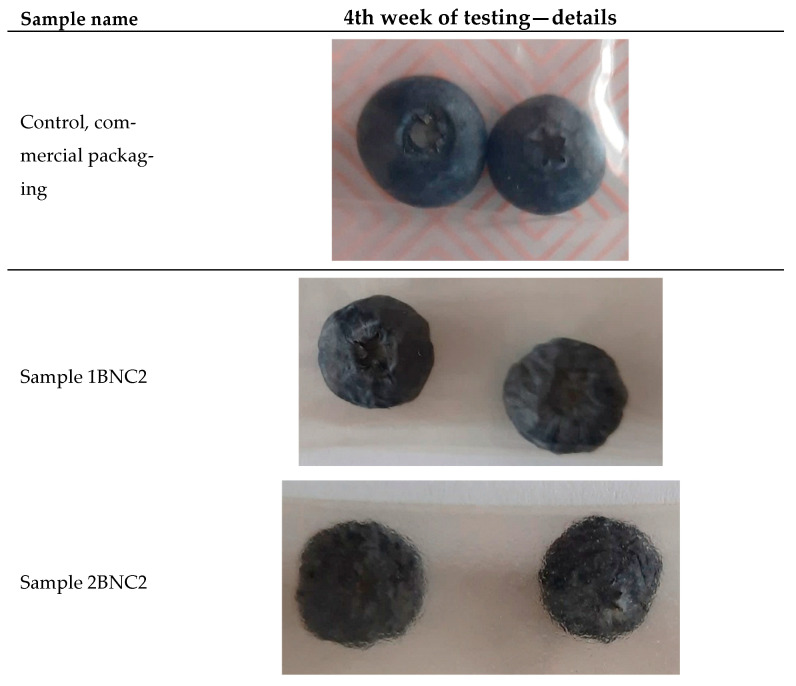
Results and details of final 4th week of testing for commercial packaging, sample with 1% of BNC/2 GSE (sample 1BNC2) and 2% of BNC/2 GSE (sample 2BNC2).

Visual assessment over four weeks revealed that blueberries in GSE films maintained their brightness, firmness, and absence of mold or microbial growth (Figure 2 and Figure 3). In contrast, control berries showed significant spoilage indicators, including mold formation, shriveling, and color degradation, especially after week three. Berries in film packages with GSE retained a fresher appearance, demonstrating the dual role of physical barrier and bioactive compounds in extending shelf life. The limitation of this study is that the films were evaluated only on blueberries, and while similar benefits are anticipated for other high-moisture foods, further validation is required for different food categories, particularly lipid-rich products.

## 4. Conclusions

This study demonstrated that the incorporation of BNC and GSE into sodium alginate-based films significantly enhanced their mechanical, barrier, and functional properties. The addition of BNC reinforced the alginate matrix, as shown by increased tensile strength and reduced water vapor permeability, confirming its role as an effective structural and barrier component. Beyond structural reinforcement, the incorporation of GSE imparted strong bioactive functionality. Antioxidant activity, measured through free radical scavenging activity and total phenolic content, increased proportionally with GSE concentration. Likewise, antimicrobial assays against Escherichia coli and Staphylococcus aureus confirmed concentration-dependent inhibitory effects, with *S. aureus* showing greater sensitivity, consistent with known differences between Gram-positive and Gram-negative bacterial cell wall structures. Among the tested formulations, the film containing 1% GSE exhibited the most favorable balance of properties. It combined mechanical reinforcement and reduced water vapor permeability with strong antioxidant and antimicrobial activity, making it the most suitable candidate for active food packaging applications where both moisture control and microbial inhibition are critical for shelf-life extension.

Nevertheless, challenges remain regarding the long-term stability of GSE in the film matrix, potential sensory effects on packaged food, and the scalability of the production process. These aspects require further investigation through extended storage trials, migration and sensory studies, and industrial validation. Despite these limitations, GSE-enriched sodium alginate/BNC films represent a promising class of multifunctional, bio-based packaging materials. By combining mechanical reinforcement, barrier enhancement, antioxidant activity, and antimicrobial performance, they provide a sustainable and effective strategy to reduce food spoilage, extend the shelf life of perishable products, and contribute to a reduction in food waste.

## Figures and Tables

**Table 1:** Sample names and combination of concentrations.

Sample Name	% of BNC (*w*/*w*)	% of GSE (*w*/*w*)	% of Glycerol (*w*/*w*)
Sample 1BNC0	1	0	15
Sample 1BNC0.5		0.5	15
Sample 1BNC1		1	15
Sample 1BNC2		2	15
Sample 2BNC0	2	0	15
Sample 2BNC0.5		0.5	15
Sample 2BNC1		1	15
Sample 2BNC2		2	15

**Table 2:** Results of thickness and mechanical properties of films.

Sample Name	Thickness [mm]	Tensile Strength [MPa]	Elongation at Break [%]	Porosity [mL/min]
Sample 1BNC0	0.085 ± 0.003	25.2 ± 1.4	15.8 ± 0.9	0
Sample 1BNC0.5	0.087 ± 0.002	28.5 ± 1.2	14.2 ± 0.4	0
Sample 1BNC1	0.089 ± 0.003	30.1 ± 1.5	14.0 ± 1.0	0
Sample 1BNC2	0.092 ± 0.004	31.7 ± 1.5	12.4 ± 0.5	0
Sample 2BNC0	0.090 ± 0.001	26.8 ± 1.2	14.7 ± 0.2	0
Sample 2BNC0.5	0.092 ± 0.002	28.9 ± 1.3	13.9 ± 0.1	0
Sample 2BNC1	0.097 ± 0.001	33.4 ± 1.6	12.7 ± 0.5	0
Sample 2BNC2	0.099 ± 0.003	39. 7 ± 1.0	12.1 ± 0.2	0

**Table 3:** Results of water vapor permeability for all prepared samples.

Sample Name	Water Vapor Permeability [g·mm/m^2^·h·kPa]
Sample 1BNC0	4.3 ± 0.1
Sample 1BNC0.5	3.7 ± 0.1
Sample 1BNC1	3.1 ± 0.2
Sample 1BNC2	2.8 ± 0.4
Sample 2BNC0	4.3 ± 0.2
Sample 2BNC0.5	3.6 ± 0.1
Sample 2BNC1	3.2 ± 0.2
Sample 2BNC2	2.9 ± 0.3

**Table 4:** Antibacterial activity of prepared film samples against *E. coli* and *S. aureus*.

Sample Name	Diameter of Bacteriostatic Circle [mm]	
	*Escherichia coli*	*Staphylococcus aureus*
Sample 1BNC0	5.8 ± 0.0	6.0 ± 0.0
Sample 1BNC0.5	8.4 ± 0.2	9.2 ± 0.1
Sample 1BNC1	12.8 ± 0.6	14.4 ± 0.1
Sample 1BNC2	19.3 ± 0.3	20.7 ± 0.4
Sample 2BNC0	6.0 ± 0.0	6.1 ± 0.0
Sample 2BNC0.5	8.0 ± 0.4	9.0 ± 0.6
Sample 2BNC1	12.4 ± 0.1	13.6 ± 1.1
Sample 2BNC2	18.1 ± 0.5	19.7 ± 0.2

**Table 5:** Results of blueberry weight loss after 4 weeks, packed in prepared films.

Sample Name	1st Week	2nd Week	3rd Week	4th Week
	Weight Loss of Packed Blueberries [%]			
Control—no film	6.8	9.4	10.5	11.3
Sample 1BNC0	1.2	3.8	3.9	4.6
Sample 1BNC0.5	1.0	3.2	3.8	4.3
Sample 1BNC1	0.9	2.8	3.8	4.0
Sample 1BNC2	0.8	2.5	3.2	3.9
Sample 2BNC0	1.3	4.0	4.4	4.4
Sample 2BNC0.5	1.1	3.4	3.5	4.0
Sample 2BNC1	1.1	3.1	3.8	4.0
Sample 2BNC2	1.0	2.8	3.3	3.8

## Data Availability

Data are contained within the article.
